# Circadian Rhythm Disturbances in Mood Disorders: Insights into the Role of the Suprachiasmatic Nucleus

**DOI:** 10.1155/2017/1504507

**Published:** 2017-11-05

**Authors:** Chelsea A. Vadnie, Colleen A. McClung

**Affiliations:** Department of Psychiatry, University of Pittsburgh Medical School, Pittsburgh, PA, USA

## Abstract

Circadian rhythm disturbances are a common symptom among individuals with mood disorders. The suprachiasmatic nucleus (SCN), in the ventral part of the anterior hypothalamus, orchestrates physiological and behavioral circadian rhythms. The SCN consists of self-sustaining oscillators and receives photic and nonphotic cues, which entrain the SCN to the external environment. In turn, through synaptic and hormonal mechanisms, the SCN can drive and synchronize circadian rhythms in extra-SCN brain regions and peripheral tissues. Thus, genetic or environmental perturbations of SCN rhythms could disrupt brain regions more closely related to mood regulation and cause mood disturbances. Here, we review clinical and preclinical studies that provide evidence both for and against a causal role for the SCN in mood disorders.

## 1. Introduction

Circadian rhythm disruptions are a major hallmark of mood disorders. Dampened and phase-shifted temperature, activity, and hormonal rhythms are frequently reported in major depressive disorder (MDD) and bipolar disorder (as reviewed in [1–4]). Studies link both environmental and genetic circadian rhythm disruptions with mood disorders. Disrupting circadian rhythms by shift work or jet lag can worsen or cause mood symptoms [5–7]. Furthermore, seasonal changes in day length can affect mood [8]. In terms of genetic disruptions, many circadian genes have been associated with mood disorders [9–13]. Since treatments that directly target the circadian system are used as therapies for mood disorders (e.g., light and dark therapies, agomelatine, social rhythm therapy, and sleep phase advance), correcting circadian disruptions may stabilize a mood [14–17].

Thus, one theory to explain the presence of circadian rhythm disruptions in mood disorders is that disrupted circadian rhythms in the master pacemaker, or suprachiasmatic nucleus (SCN), cause mood disturbances. Alternatively, some studies suggest that light directly impacts other brain regions, independent of the SCN, to control mood [18]. A third viewpoint is that sleep and circadian rhythm changes are a symptom of mood disorders and are not causal. Here, we discuss preclinical and clinical work that provide insight into whether there may be a role for the SCN in mood regulation.

## 2. Circadian Rhythms and the Central Clock

Circadian rhythms are endogenous processes with an approximate 24 hr cycle. At the cellular level, circadian rhythms are generated by a molecular clock that consists of multiple transcriptional/translational negative feedback loops (as reviewed in [19]). The positive arm of the core molecular clock consists of the transcription factors CLOCK and BMAL1, which heterodimerize and regulate the expression of many clock-controlled genes. Notably, CLOCK/BMAL1 drives the expression of *Period* (*Per1*, *Per2*, and *Per3*) and *Cryptochrome* (*Cry1*, *Cry2*), which make up the negative arm of the core molecular clock. PER and CRY heterodimerize and enter the nucleus, where they can inhibit their own transcription. When PER and CRY levels become low, CLOCK/BMAL1 then reinitiate transcription of *Per* and *Cry*. The timing of this molecular clock is regulated by numerous kinases (e.g., casein kinase 1, CK1, and glycogen synthase kinase-3, GSK-3), phosphatases, and ubiquitin ligases (e.g., FBXL3) that affect the heterodimerization and degradation of PER and CRY (as reviewed in [20]). In addition to this core transcriptional/translational feedback loop, there is a secondary feedback loop involving the orphan nuclear receptors, REV-ERB*α* and ROR*α* [20]. CLOCK/BMAL1 drives the expression of *Rev-erbα* and *Rorα*, which in turn regulate the rhythmic expression of *Bmal1*.

Although nearly all tissues express circadian genes, the SCN has several properties that make it capable of driving and synchronizing rhythms. One property is that the SCN consists of self-sustaining oscillators. When the SCN is isolated, it exhibits persistent, robust electrical and molecular rhythms (as reviewed in [21]). At the tissue level, SCN firing frequency is high during the day and low at night [21]. Thus, in diurnal animals, the peak in SCN activity occurs when the animals are active, whereas in nocturnal animals, the peak in SCN activity occurs when the animals are inactive. Progress has been made in identifying many of the ion channels underlying spontaneous SCN neural activity (as reviewed in [22]).

Another important property of the SCN is that some SCN neurons can directly respond to external cues, or zeitgebers. One powerful external cue is light. The SCN receives light information from glutamatergic projections from intrinsically photosensitive retinal ganglion cells (ipRGCs) [23, 24]. When animals are free-running in constant darkness, light has little phase-shifting effects during the middle of the subjective day, when SCN activity is high [25]. However, exposure to light during the subjective night phase shifts SCN neural activity and bodily rhythms (as reviewed in [26]). Specifically, glutamate from ipRGCs acts on NMDA and AMPA receptors on retinorecipient SCN neurons to increase neural activity and activate cellular signaling. Early in the subjective night, when PER levels are decreasing, ipRGC signaling increases *Per* expression, inducing a phase delay. Late in the subjective night, when *Per* expression is starting to increase, ipRGC signaling induces an increase in *Per* expression, promoting a phase advance. The SCN also responds to nonphotic cues, such as behavioral arousal (as reviewed in [27]).

A unique property of the SCN is the SCN network, which allows for robust, synchronized SCN neuronal rhythms (as reviewed in [28]). The SCN is a heterogenous tissue with a complex network. The majority of SCN neurons are GABAergic and secrete different peptide neurotransmitters. The peptide neurotransmitters are expressed in distinct regions of the SCN, indicating that they have different functional roles. Many of these distinct SCN neurons exhibit electrical and molecular rhythms when isolated, but the rhythms are weaker and unstable [29–31]. Thus, the intrinsic SCN network appears to be important for generating robust, synchronized SCN oscillations. Numerous mechanisms have been implicated in the coupling of SCN neurons, including specific neuropeptides, gap junctions, astrocytes, and GABAergic signaling (as reviewed in [28, 32]). Vasoactive intestinal peptide (VIP) and arginine vasopressin (AVP) are two of the more well-studied neuropeptides involved in regulating SCN rhythms. Studies indicate that VIP is necessary to maintain and synchronize rhythms in the SCN [33, 34], whereas AVP is involved in maintaining high amplitude output from the SCN and in modulating SCN re-entrainment [35–37].

**Figure 1:**
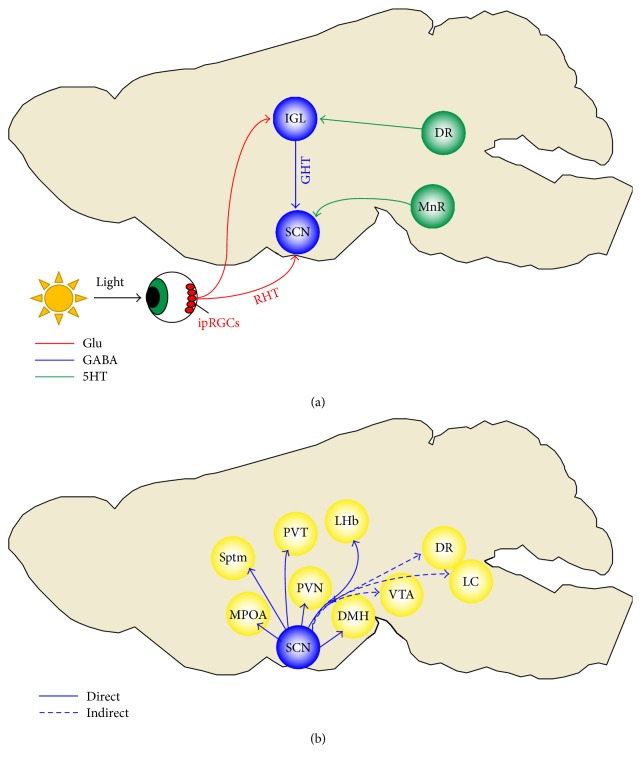
Inputs and outputs of the suprachiasmatic nucleus (SCN). (a) The main inputs to the SCN come from the intrinsically photosensitive retinal ganglion cells (ipRGCs), median raphe (MnR), and intergeniculate leaflet (IGL) (as reviewed in [38]). The retinohypothalamic tract (RHT) originates from ipRGCs and primarily terminates in the SCN. The RHT terminals release glutamate (Glu) and pituitary adenylate cyclase-activating polypeptide, which entrain the SCN to the light-dark cycle [379, 380]. ipRGCs also project to the IGL [381]. The pathway from the IGL to the SCN is called the geniculohypothalamic tract (GHT). GHT terminals release GABA and neuropeptide Y onto the SCN (as reviewed in [382]). GHT relays photic and nonphotic information to the SCN. The SCN also receives input from midbrain raphe nuclei, directly from the MnR and indirectly from the dorsal raphe (DR) through the IGL [383]. Serotonergic (5HT) signaling in the SCN modulates the effects of photic cues and plays a major role in the effects of nonphotic cues [130, 131]. (b) The SCN projects to other areas of the hypothalamus, including the paraventricular nucleus (PVN), dorsomedial nucleus (DMH), and the medial preoptic area (MPOA) (as reviewed in [38]). The SCN also directly projects to areas outside of the hypothalamus, such as the paraventricular nucleus of the thalamus (PVT), septum (Sptm), and lateral habenula (LHb). The SCN indirectly projects to the ventral tegmental area (VTA), locus coeruleus (LC), and DR [38, 43].

The SCN network is also essential for integrating afferent signals and generating synchronized bodily rhythms. Tract tracing studies have identified many of the SCN inputs and outputs (as reviewed in [38]). The main SCN inputs come from ipRGCs, the median raphe, and intergeniculate leaflet, which relay information about photic and nonphotic cues (Figure 1(a)). Transplant studies have revealed that the SCN sustains circadian rhythms by both synaptic connections and hormonal mechanisms [39, 40]. In terms of direct outputs, the SCN mainly projects to other hypothalamic regions, such as the dorsomedial nucleus (DMH), paraventricular nucleus (PVN), and the medial preoptic area (MPOA) (as reviewed in [38]). The SCN also projects to regions outside of the hypothalamus, such as the paraventricular zone of the thalamus (PVT) and septum (Figure 1(b)). Some studies suggest that SCN directly projects to the lateral habenula, but this is still debatable [41, 42]. Furthermore, multisynaptic pathways from the SCN have been identified [38, 43]. Specifically, the SCN indirectly projects to the locus coeruleus, ventral tegmental area, and dorsal raphe, suggesting mechanisms through which the SCN could regulate arousal, reward, and mood.

## 3. Circadian Rhythm Disturbances in Humans with Mood Disorders

### 3.1. Circadian Rhythm Disturbances in Depression

Decades of research have pointed out associations between rhythm disturbances and depression (Table 1). Sleep disruptions are a commonly reported circadian-related disturbance in depression (as reviewed in [1]). It should be noted that although sleep timing is regulated by the circadian system, sleep is a complex biological process that is also regulated by homeostatic mechanisms. Currently, sleep-wake disruptions are included in the diagnostic criteria for MDD. Individuals with typical depression frequently report early morning awakening and disrupted sleep (as reviewed in [1]). Conversely, in atypical depression, individuals often have later sleep times and sleep longer, but experience daytime fatigue. Moreover, hypersomnia and insomnia are associated with greater suicidality, emphasizing the importance of treating sleep disturbances in depression [44]. More specifically, studies indicate that reduced latency to REM sleep, increased REM time, and decreased slow-wave sleep frequently occur in depression [45–48].

In the 1980s, it was proposed that the phase of rhythms tightly controlled by the central clock is disturbed in depression (as reviewed in [4]). Specifically, multiple studies pointed to a phase advance in hormonal rhythms in depression [48–50]. However, more recent studies indicate that rhythms are delayed in depression [51–55]. Supporting the phase delay hypothesis, as discussed later, some SSRIs speed up rhythms [56]. Moreover, early morning bright light therapy, which induces phase advances, can be effective in reducing symptoms of seasonal and nonseasonal depression [57, 58]. Furthermore, delayed sleep phase syndrome and westward travel (induces phase delays) can increase vulnerability to depression [5, 59, 60].

Studies also show that rhythm amplitude is dampened in depression. Reduced body temperature amplitude and increased nocturnal body temperature are frequently found in depression [50, 61, 62]. Studies also reported dampened activity, cortisol, thyroid-stimulating hormone, melatonin, and heart rate rhythms in depression [50, 63–65]. Interestingly, there are some reports of rhythm amplitude increasing as patients recover, suggesting that enhancing rhythms may be therapeutic [50, 66].

There is also evidence to support that molecular rhythms are disrupted in depression. A postmortem study by Li and colleagues used a time-of-death analysis to determine whether there were differences in gene expression rhythms in six mood-related brain regions (anterior cingulate, dorsolateral prefrontal cortex, hippocampus, amygdala, nucleus accumbens, and cerebellum) in subjects with depression [67]. Out of 12,000 transcripts, hundreds of rhythmic genes were identified in control subjects. The genes with the most robust rhythms were clock genes. Remarkably, many circadian genes were not rhythmic in depressed patients. Furthermore, by comparing gene expression between controls and depressed patients that died at similar times, it was concluded that gene expression rhythms were likely phase shifted and desynchronized from external time in depression. Li and colleagues also looked at genes thought to be in-phase and out-of-phase in controls versus depressed subjects. Phase relationships held up in controls, but were disrupted in depressed subjects, indicating that gene expression rhythms were desynchronized from one another in individual brain region in depression.

It is possible that perturbations in circadian genes underlie some of the circadian rhythm disturbances observed in depression. Genetic studies have implicated many circadian genes in MDD. These genes include *CRY1*, *NPAS2*, *NR1D1* (*REV-ERBα*), and others (as reviewed in [68, 69]). However, many of the findings need to be replicated in larger sample sizes.

### 3.2. Circadian Rhythm Disturbances in Bipolar Disorder

Bipolar disorder is characterized by reoccurring episodes of mania with or without episodes of depression. As with MDD, sleep disturbances frequently occur in bipolar disorder and are part of the diagnostic criteria for bipolar disorder. Specifically, there is typically reduced sleep during manic episodes, and insomnia or hypersomnia during depressive episodes (as reviewed in [70]). There have been mixed findings on how sleep architecture is affected in bipolar disorder. The most consistent finding is reduced REM latency and increased REM density in mania, suggesting that there is not a “decreased need for sleep” during mania as stated in the DSM-5, but an inability to obtain sufficient sleep [71–73]. Sleep disturbances are also prevalent during euthymia, indicating that sleep is still affected after mood has stabilized [74]. Although sleep disturbances are present in between mood episodes, sleep disturbances worsen just before relapse and more so during mood episodes, again highlighting the need for treatments for sleep disturbances [75, 76].

Dampened and shifted circadian rhythms may explain some of the sleep disturbances frequently found in and reported by patients with bipolar disorder. Actigraphy studies have revealed less rhythmic activity and dampened activity amplitude in bipolar disorder [77–79]. Others have reported dampened body temperature and hormonal rhythms in bipolar disorder [80]. In addition, one of the most commonly reported rhythm-related findings in bipolar disorder is an evening chronotype [81–83]. There have been mixed findings on whether metabolite, sleep-wake, body temperature, and hormone rhythms are delayed or advanced in bipolar disorder [84–87]. One explanation is that the phase changes in rhythms may be state dependent. Some studies suggest that rhythms are advanced during mania, delayed during depressive episodes, and more entrained when patients reach a euthymic state [88–90]. Particularly interesting is the study by Moon and colleagues showing that clock gene expression rhythms from buccal cells and cortisol rhythms from saliva were mostly advanced during manic episodes and delayed during depressive episodes in hospitalized bipolar patients relative to controls [88]. Moon and colleagues then showed that rhythms are delayed in the previously manic patients and advanced in the previously depressed patients during recovery, suggesting that buccal cell clock gene and saliva cortisol rhythms could be used as state markers. Conversely, hypersensitivity to light-induced suppression of melatonin has been proposed to be a trait marker for bipolar disorder [91, 92], but other studies have found no difference in light-induced melatonin suppression in patients with bipolar disorder [87, 93].

As with MDD, genetic studies have implicated clock genes in bipolar disorder (see [94]). These genes include *CLOCK*, *BMAL1*, *PER3*, *NR1D1*, and others. There is a high interest in identifying genetic risk factors for bipolar disorder since the heritability is estimated to be as high as 85% [95]. However, whether disruptions in these genes are risk factors for bipolar disorder is still controversial, as many of these findings have not been replicated.

For a comprehensive discussion of circadian rhythm disruptions in depression and bipolar disorder, see the following reviews [1–4, 96, 97].

### 3.3. The Central Clock in Mood Disorders

A few studies suggest that SCN function is perturbed in mood disorders. Zhou and colleagues found increased AVP-immunoreactive cells, but decreased AVP mRNA in the SCN of subjects with depression or bipolar disorder relative to controls [98]. Zhou and colleagues interpreted the results as suggesting that AVP transport and release is decreased in the SCN of subjects with mood disorders, which results in a buildup of AVP in SCN neurons. These findings were corroborated by a later study from the same laboratory. In this study, the number of cells that was AVP and/or VIP-immunoreactive was increased in the SCN of individuals with major depression or bipolar disorder [99]. Interestingly, Wu and colleagues also found that the number of AVP and/or VIP-immunoreactive cells was positively correlated with disease duration and negatively correlated with the age of onset. Thus, SCN AVP and VIP signaling may be altered in mood disorders.

Nitric oxide signaling in the SCN may also be affected in mood disorders. Nitric oxide synthase (NOS)-immunoreactive neurons were found to be reduced in the SCN of patients with depression or bipolar disorder relative to controls [100]. Nitric oxide signaling in the SCN conveys photic information, and thus individuals with mood disorders may exhibit disrupted nitric oxide signaling-dependent responses to changes in the light-dark cycle [101]. Preclinical studies have implicated nitric oxide signaling in mood regulation [102]. Thus, altered nitric oxide signaling may affect both mood and circadian rhythms.

Disrupted melatonin feedback onto the SCN may also occur in mood disorders. Wu and colleagues found increased melatonin type 1 receptor (MT1)-immunoreactive cells in the SCN of subjects with depression or bipolar disorder [99]. In this same study, the number of SCN MT1-immunoreactive cells was positively correlated with the duration of disease and negatively correlated with the age of onset [99]. One possible explanation, provided by the authors, was that the increase in SCN MT1-immunoreactive cells was a compensatory response due to potentially low levels of melatonin in the subjects with mood disorders. However, melatonin levels were not measured in this study. Furthermore, low melatonin levels have not been consistently reported in mood disorders [103].

Although there are no reports of SCN structural abnormalities in mood disorders, there is evidence of reduced hypothalamic volume and dilation of the third ventricle in mood disorders [104, 105]. Furthermore, some neuroanatomical differences have been found in direct targets of the SCN. Several studies showed alterations in the size and function of the PVN in subjects with mood disorders [106–110]. Functionally, the SCN to PVN circuit is important for the control of pituitary hormones and melatonin secretion from the pineal gland (as reviewed in [111]). If the SCN to PVN circuit is disrupted in mood disorders, this may explain alterations in hormone rhythms. The habenula, another possible direct output of the SCN, has been implicated in mood and circadian regulation. Some studies show reduced habenula volume, but other studies have found opposite or no difference in habenula volume in subjects with mood disorders [112–115]. Notably, deep brain stimulation (DBS) of the main lateral habenula afferent reduced depressive symptoms in several patients [116, 117]. Based on animal studies, it is hypothesized that DBS of the main lateral habenula afferent reduced depressive symptoms in these small studies by suppressing lateral habenula activity. Several animal models exhibiting depression-like behavior show increased habenula metabolism [118, 119]. Moreover, the antidepressant, fluoxetine, reduced lateral habenula metabolism in rats [120]. Preclinical studies also show that lesioning the lateral habenula can reduce depression-like behaviors [121, 122]. The lateral habenula is an intriguing putative target of the SCN since it exhibits intrinsic neuronal and molecular oscillations [123, 124]. Moreover, some lateral habenula neurons respond to retinal illumination [123], presumably through indirect neuronal connections, such as through the SCN, since ipRGCs preferentially project to the parahabenula [125]. The lateral habenula is known to modulate monoaminergic nuclei activity, thus the lateral habenula could act as a relay between the SCN and brain regions more closely involved in mood regulation (as reviewed in [126]).

## 4. Effects of Pharmacotherapies on the SCN

### 4.1. SSRIs

Serotonin reuptake inhibitors (SSRIs) have remained a first-line treatment for MDD, but as revealed by the Sequenced Treatment Alternatives to Relieve Depression (STAR^∗^D) trial, the majority of patients do not achieve remission after treatment with an SSRI [127]. Thus, there is a need to better understand the mechanisms underlying SSRI efficacy. There are numerous interactions between the circadian and serotonergic systems, suggesting that the effects of SSRIs on circadian rhythms could influence efficacy (as reviewed in [128]). The SCN receives serotonergic projections from the median raphe nucleus, and in turn, the SCN indirectly projects to midbrain raphe nuclei [43, 129]. Serotonin levels peak in the SCN during the active phase of mammals [130, 131]. In the SCN, serotonin plays a key role in the phase-shifting effects of nonphotic cues (e.g., behavioral arousal). Specifically, serotonin receptor antagonists reduced the phase-advancing effects of behavioral arousal during the inactive phase of nocturnal animals, indicating that serotonin signaling in the SCN is involved in the phase-advancing effects of nonphotic cues [132]. Furthermore, serotonin or serotonin receptor agonists induced phase advances in SCN neural activity *in vitro* and in behavioral activity rhythms when administered in the SCN [133–136]. Not surprisingly, SSRIs have similar effects on circadian rhythms (Table 2). The SSRI, fluoxetine, phase advanced SCN neural activity rhythms [56, 137]. Fluoxetine also phase advanced locomotor activity rhythms in both nocturnal and diurnal animals [130, 131]. Overall, these studies suggest that SSRIs could treat a delayed component of circadian rhythms in depression.

Few studies have examined the effects of chronic SSRI treatment on circadian rhythms in rodents. Some studies show no effects of chronic SSRI treatment on the phase of entrained rhythms or the period of free-running rhythms [138–140]. Conversely, it has been reported that chronic fluoxetine treatment shortens the period of locomotor activity rhythms in mice [141, 142], which is supported by the finding that the SSRI sertraline shortened the period of PER1::LUCIFERASE (PER1::LUC) bioluminescent rhythms in the mouse SCN [143]. In future studies, it will be important to confirm whether chronic SSRI treatment affects circadian period and other circadian measures in various animal models.

The slow-acting effects of antidepressants on mood may be related to a gradual restoration of circadian rhythms by chronic antidepressant treatment. Since chronic SSRI treatment decreases and desensitizes serotonin receptors, it may affect SCN synaptic plasticity (as reviewed in [144]). Several serotonin receptor subtypes expressed in the SCN are known to have circadian-related effects (as reviewed in [131]). Thus, chronic use of SSRIs may modulate circadian rhythms through affecting the expression or availability of specific subtypes of serotonin receptors in the SCN. Supporting this theory, fluoxetine reduced the expression of 5HT_1B_ receptors in the SCN of aged Syrian hamsters [140]. Another intriguing hypothesis is that chronic SSRI use may affect circadian rhythms and mood through brain-derived neurotrophic factor (BDNF)-tropomyosin-related receptor kinase (TrkB) signaling in the SCN. There is evidence to support that SSRI-mediated restoration of BDNF-TrkB signaling in the brain partially underlies the antidepressant-like effects of SSRIs in animal models of depression (as reviewed in [145, 146]). Interestingly, TrkB receptors in the SCN are known to regulate photic phase shifts [147–149]. Thus, it is possible that chronic SSRI treatment facilitates the resynchronization of misaligned rhythms in individuals with depression through BDNF-TrkB signaling in the SCN.

### 4.2. Lithium

Lithium, a commonly prescribed mood stabilizer, is known to affect multiple aspects of circadian rhythms. One well-established effect of lithium is lengthening circadian period. Lithium lengthens the period of rhythms in a wide range of species, including insects, nocturnal animals, diurnal animals, humans, and plants [150–157]. In animals, lithium likely increases the period of behavioral and physiological rhythms through its actions in the SCN. Lithium lengthens the period of SCN neural activity and SCN PER2::LUC bioluminescent rhythms [153, 156, 158–160]. Lithium also increases the amplitude of PER2::LUC rhythms in the SCN, suggesting that lithium enhances the amplitude of physiological and behavioral rhythms as well [153, 156]. Thus, lithium may correct phase-advanced and dampened rhythms in patients with mood disorders, which could contribute to the mood-stabilizing effects of lithium. Indeed, there is some evidence to support this since a study by Kripke and colleagues showed that individuals with bipolar disorder that responded to lithium had faster rhythms than nonresponders before treatment [157].

One well-studied target of lithium that may explain some of its circadian-related effects is GSK-3. GSK-3 is a serine/threonine kinase consisting of two paralogs, GSK-3*α* and GSK-3*β*. GSK-3 is different from typical kinases in that it is usually active and regulated by phosphorylation of inhibitory serine residues [161]. Lithium directly and indirectly inhibits GSK-3 (as reviewed in [162]). Pharmacological and genetic studies in animal models indicate that the effects of lithium on amplitude may be explained by inhibition of GSK-3, but GSK-3 does not appear to be involved in the effects of lithium on rhythm period. Specifically, GSK-3 inhibitors increase the amplitude of circadian rhythms like lithium, but shorten circadian period, unlike lithium [153, 163]. Mice that express GSK-3*α* and GSK-3*β* with serine residues that have been mutated to block GSK-3 inhibition (GSK-3 knockin, GSK-3 KI mice) have decreased wheel-running rhythm amplitude and, on a mixed background, have a longer free-running activity period [164]. These effects seem to be mediated by GSK-3 in the SCN. Inhibition of GSK-3 was shown to reduce the period and increase the amplitude of PER2::LUC rhythms in SCN explants [165]. Moreover, GSK-3 KI mice exhibited increased SCN firing frequency during the night, indicating that chronically increased GSK-3 activity reduces the day/night difference in SCN neural activity [164].

Preclinical studies indicate that GSK-3 also regulates mood-like behaviors. The most consistent finding is that GSK-3 regulates mania-like hyperactivity. Amphetamine administration, at doses that induce hyperactivity, decrease the inhibitory serine phosphorylation on GSK-3 [166, 167]. Inhibiting GSK-3 suppresses the effects of psychostimulants, while increasing GSK-3 activity enhances the effects of psychostimulants [168, 169]. Moreover, GSK-3 KI and GSK-3*β* overexpressing mice are hyperactive [169, 170]. Conversely, GSK-3*α* KO mice are hypoactive [171]. Genetic and pharmacological studies also suggest that GSK-3 regulates depression and anxiety-like behaviors. GSK-3*α* KO mice and GSK-3*β*^+/−^ mice show reduced depression-like behavior [171, 172]. Conversely, GSK-3 KI mice exhibit increased anxiety- and depression-like behaviors [169]. However, these results conflict with the conclusion that increased GSK-3 activity results in mania-like hyperactivity. One interpretation by Polter and colleagues is that elevated GSK-3 activity increases sensitivity to stress and may therefore induce depression or mania-like behavior, depending on the environment [169].

GSK-3 phosphorylates several key components of the molecular clock (e.g., PER2, CRY1, CLOCK, BMAL1, and REV-ERB*α*), which points to mechanisms by which lithium may regulate the timing of the SCN and molecular rhythms in mood-related brain regions [173–177]. GSK-3 phosphorylates PER2, promoting its nuclear translocation and degradation [174]. Thus, lithium could increase the amplitude of PER2 rhythms through inhibition of GSK-3. It is controversial whether GSK-3 is involved in the period lengthening effects of lithium, as previously discussed. Genetic variants in other genes (i.e., *CACNA1C*, *RORA*, and *PER3*) have been implicated in the effects of lithium on rhythm amplitude or period, indicating that lithium may affect circadian rhythms through direct or indirect actions on other clock genes [178, 179].

### 4.3. Valproic Acid

Valproic acid, an anticonvulsant used as a treatment for mania, also affects the central clock. Valproic acid increased the amplitude of PER2::LUC rhythms in the mouse SCN [160]. Valproic acid induced phase advances or delays in PER2::LUC rhythms in the mouse SCN depending on the timing of drug application [160]. Past studies reported inconsistent effects of valproic acid on circadian period [160, 180–182]. A recent study by Landgraf and colleagues presented convincing evidence that valproic acid has opposing effects on circadian period compared to lithium [183]. Valproic acid shortened the period of mouse wheel-running activity, PER2::LUC rhythms in mouse SCN explants, PER2::LUC rhythms in mouse hippocampal cell culture, and PER2::LUC rhythms of cultured human fibroblasts from patients with bipolar disorder [183].

The ability of valproic acid to inhibit GSK-3, or inhibit class I HDACs (which bind to CLOCK and BMAL1), could potentially explain both the mood-stabilizing and circadian effects of valproic acid (as reviewed in [184, 185]). Preclinical studies support that GSK-3 inhibitors and possibly HDAC inhibitors have mood-stabilizing effects [166, 186]. In terms of circadian rhythms, it was shown that the HDAC inhibitor trichostatin A induced phase shifts and an enhancement of SCN PER2::LUC rhythms similar to valproic acid [160]. It is known that there are rhythms in the acetylation of clock proteins and in histone acetylation at clock gene promoters [187, 188]. Thus, HDAC inhibition by valproic acid could affect circadian rhythms by increasing the acetylation of the molecular clock. On the other hand, GSK-3 inhibitors shorten circadian period, which could also explain the period-shortening effects of valproic acid [153, 163, 183]. Future studies aimed at uncovering the molecular mechanisms of the effects of valproic acid have potential to lead to new therapeutics for mood disorders and increase our understanding of the biology of circadian regulation.

### 4.4. Melatonin

The SCN regulates melatonin secretion from the pineal gland through a multisynaptic pathway involving the PVN (as reviewed in [111]). The SCN inhibits melatonin synthesis during the day and stimulates melatonin production at night [189]. Thus, melatonin levels are the highest at night in both nocturnal and diurnal animals. Melatonin exerts most of its effects via the MT1 and MT2 G protein-coupled melatonin receptors, which are widely expressed in the brain (including the SCN) and peripheral tissues [190–192]. Several studies suggest that the nightly release of melatonin is involved in regulating rhythms in other bodily tissues [193–195].

Melatonin also feeds back onto the SCN, which is thought to underlie the circadian-related effects of melatonin. In rodents and humans, melatonin induces phase advances when given at the light-to-dark transition and, to a lesser extent, phase delays when administered at the dark-to-light transition [196–198]. Melatonin also phase shifts SCN neural activity rhythms at dusk and dawn [199–201]. Moreover, melatonin suppresses SCN neural activity and increases the amplitude of physiological rhythms, indicating that melatonin can enhance rhythms through its actions in the SCN [202–205]. Melatonin also entrains circadian rhythms; an action that is also dependent upon the SCN. Specifically, daily administration of melatonin at the light-to-dark transition entrained free-running rhythms in rodents [206]. Melatonin did not entrain the free-running rhythms of SCN-lesioned rodents, indicating that the SCN is necessary for the entraining effects of melatonin [206]. Together, these studies suggest that melatonin may be a useful adjunct therapy for treating circadian disruptions in mood disorders.

Dampened or phase-shifted melatonin rhythms have been reported in mood disorders, especially depression [207–212]. Preclinical studies indicate that melatonin has antidepressant-like effects [213–217]. However, in humans, there is no evidence to support that melatonin is an effective treatment for depression [218].

Agomelatine, a synthetic melatonin analog, also produces antidepressant-like effects in preclinical studies [219, 220]. Studies suggest that the synergistic actions of agomelatine at both MT1/MT2 and 5HT_2C_ receptors underlie the antidepressant-like effects of agomelatine in animal models (as reviewed in [221, 222]). Agomelatine was approved in 2009 as a treatment for MDD in the European Union, but it is controversial whether agomelatine is more efficacious than other antidepressants [223]. Several studies showed that agomelatine improved sleep in individuals with depression, suggesting that agomelatine may be particularly useful for sleep disturbances in depression [224–226]. However, it is unclear whether the sleep/circadian effects of agomelatine contribute to its efficacy as an antidepressant.

Since agomelatine is a MT1/MT2 receptor agonist, it is not surprising that agomelatine similarly affects circadian rhythms. Agomelatine also induces phase shifts, decreases SCN neural firing, entrains rhythms, and accelerates re-entrainment [204, 227–233]. Similarly, the SCN is thought to be the site of action for the circadian-related effects of agomelatine [234]. Although differing from melatonin, agomelatine acts as a 5HT_2C_ receptor antagonist. 5HT_2C_ receptors are expressed in the SCN, and studies indicate that 5HT_2C_ receptors play a role in some of the circadian-related effects of agomelatine [235]. Specifically, the 5HT_2C_ receptor agonist, Ro60-0175, was shown to reduce the ability of agomelatine to suppress SCN neural firing, indicating that inhibition of 5HT_2C_ receptors by agomelatine contributes to its effects on dampening SCN neural activity [231].

## 5. Effects of SCN Manipulations on Mood-Like Behaviors in Rodents

Early studies investigated whether the SCN regulates mood by assessing mood-like behaviors in SCN-lesioned rats. Two groups found that lesioning the SCN resulted in less immobility in the forced swim test [236, 237]. One interpretation is that disrupting SCN function has antidepressant effects. However, since the behavioral tests in these studies were likely carried out during the light phase, when immobility time in the forced swim test may be higher, lesioning the SCN could have ameliorated the circadian variation in mood-like behaviors [238]. A third study investigated whether lesioning the SCN affected anxiety-like behaviors of rats that had or had not experienced social defeat [239]. Tuma and colleagues found that lesioning the SCN had no effect on anxiety-like behaviors of defeated or nondefeated controls when in the presence of an enclosed aggressive rat, suggesting that the SCN does not regulate anxiety-like behavior [239]. Overall, it is difficult to draw conclusions about the role of the SCN in mood regulation from these studies since they lack information about the behavior of the animals across the day and since pathways traversing the SCN were destroyed.

To determine the role of the SCN in mood regulation in a neuroanatomically intact system, Landgraf and colleagues disrupted the SCN molecular clock by virally knocking down *Bmal1* expression in the SCN [240]. They achieved a 60% knockdown of SCN BMAL1, which resulted in a dampening and lengthening of SCN PER2::LUC rhythms. SCN BMAL1 knockdown also lengthened wheel-running rhythms. Most notably, disruption of SCN molecular rhythms increased depression-like behavior in the learned helplessness and tail suspension tests. Additionally, SCN BMAL1 knockdown increased anxiety-like behavior in the light/dark box. Together, these findings suggest that reduced amplitude and increased period of SCN molecular rhythms can cause increased depression and anxiety-like behavior.

## 6. Light Cycle Manipulations and the SCN

### 6.1. Seasonal Affective Disorder (SAD)

Seasonal changes in day length (photoperiod) affect mood. SAD is commonly characterized as reoccurring fall/winter depression with spontaneous remissions occurring in the spring/summer [8]. It has been proposed that winter depression in SAD is caused by an expansion and/or delay in the offset of melatonin secretion, driven by photoperiodic changes in SCN activity (as reviewed in [241, 242]). From rodent work, it is known that during short winter-like days the peak of SCN neuronal activity is compressed, whereas during long summer-like days the peak of SCN neuronal activity is expanded [243]. Since the SCN inhibits melatonin synthesis during the day and promotes melatonin synthesis at night, compressed SCN activity during the winter results in a lengthening of melatonin release (as reviewed in [189]). Alternatively, due to a later dawn in the winter, melatonin and other rhythms that are tightly controlled by the SCN may be delayed in SAD [244]. In humans, there have been some reports of delayed melatonin offset or increased seasonal changes in melatonin in individuals with SAD, but these changes in melatonin are not consistently observed (as reviewed in [245, 246]).

A study in rats has suggested that photoperiodic-induced neurotransmitter switching may explain the effects of photoperiod on mood-like behaviors [247]. Exposing nocturnal rats to a short photoperiod reduced anxiety- and depression-like behaviors and produced a switch from somatostatin to dopaminergic neurons in hypothalamic brain regions that receive input from the SCN. A long photoperiod had the opposite effect, increasing anxiety- and depression-like behaviors and producing a switch from dopaminergic to somatostatin neurons in the hypothalamus. Dulcis and colleagues then examined the effects of ablating dopaminergic neurons in these hypothalamic regions in combination with housing the rats in different photoperiods. Ablating hypothalamic dopaminergic neurons increased depression- and anxiety-like behaviors, resembling the effects of a long photoperiod. The mood-related effects of ablating the hypothalamic dopaminergic neurons were reduced by exposing the rats to a short photoperiod and enhanced by exposing the rats to a long photoperiod, indicating that hypothalamic neurotransmitter switching may underlie the effects of photoperiod on mood. Photoperiod-induced neurotransmitter switching may also occur in humans. A postmortem study of brains obtained from individuals from a high altitude (Scotland) showed that there was an increased number of dopaminergic neurons in the midbrain during long photoperiod months [248]. Although different brain regions, this was opposite to what was observed in rats. The opposing effects are likely due to differences in the circadian system between nocturnal and diurnal species.

Since there are fundamental differences in the circadian system of nocturnal versus diurnal animals, researchers have been moving towards using diurnal rodents for studying SAD (as reviewed in [249]). For example, melatonin has opposite effects on body temperature [250, 251] and sleep [251, 252] in nocturnal versus diurnal animals. Thus, it is not too surprising that in nocturnal rodents melatonin reduces [213, 214, 253], but in diurnal rodents melatonin increases or has no effect on anxiety- and depression-like behaviors [253, 254]. Moreover, if melatonin plays a role in the mood-related effects of photoperiods, many nocturnal mouse species would not be ideal model organisms since most strains have lost the ability to synthesize melatonin due to acquired mutations in enzymes involved in melatonin synthesis [255, 256]. It is also known that photoperiod can have opposite effects in diurnal versus nocturnal rodents. Short photoperiods compress the length of the active phase in diurnal animals, but expand the length of the active phase in nocturnal animals [257]. Regarding the effects of photoperiod on mood-like behaviors, long and short photoperiods appear to have inconsistent effects in nocturnal animals [247, 258, 259]. Conversely, increasing studies show that short photoperiods, or winter-like light schedules, increase anxiety- and depression-like behaviors in diurnal rodents (as reviewed in [260, 261]). A study by Leach and colleagues suggests that diurnal animals are more vulnerable to the mood-like effects of short photoperiods since diurnal animals are less able to adapt SCN clock gene expression and locomotor rhythms to short photoperiods relative to long photoperiods [262]. Finally, diurnal rodent models of SAD appear to respond to current treatments for depression [263, 264]. Thus, studies argue that diurnal SAD rodent models have construct, face, and predictive validities.

### 6.2. Constant Light and Constant Dark

Constant lighting conditions affect mood-like behaviors in rodents, but the role of the SCN in the effects of constant lighting on mood is unclear. Constant light and especially dim light at night models are translationally relevant to understand how exposure to artificial light at night may impact human mental health. Constant light and dim light at night increase depression-like behavior and have mixed effects on anxiety-like behavior in rodents [265, 266]. Constant bright light has greater disruptive effects on circadian rhythms relative to dim light at light. Constant light desynchronizes the molecular rhythms of neurons in the SCN [267]. As a result, constant light flattens hormonal and body temperature rhythms (as reviewed in [268]). Moreover, constant light perturbs locomotor rhythms, typically increasing period length, inducing rhythm splitting, and arrhythmia. Some studies show that dim light at night has subtle effects on homecage circadian activity rhythms and dampens SCN molecular rhythms [268, 269]. Like constant light and dim light at night, constant dark also increases depression-like behavior [270–272]. Under constant darkness, rodents still exhibit robust circadian rhythms, but do appear to show a decreased amplitude of sleep-wake rhythms [273]. The common link between these studies is that they implicate SCN amplitude in mood regulation. There is also evidence to support that constant lighting may disrupt mood by inducing monoaminergic neuron apoptosis, but it is unclear if these effects are related to SCN amplitude [271, 274].

### 6.3. Jet Lag

Jet lag can provoke or exacerbate mood disturbances in humans [5]. Shifting the light-dark cycle in rodents also affects mood-like behaviors. Five weeks of repeated phase advances, or advances and delays, increased anxiety-like behavior in mice [275]. In another study, five weeks of repeated delays reduced anxiety-like behavior in rats, suggesting that delays and advances have opposing effects on mood-like behaviors [276]. Interestingly, human studies do indicate that eastward jet lag and westward jet lag have opposing effects on mood. In individuals with mood disorders, eastward jet lag is more likely to precipitate manic episodes, whereas westward jet lag is more likely to induce depressive episodes [5, 277]. The detrimental effects of repeated phase shifts may be due to a chronic internal desynchronization of circadian rhythms. Studies indicate that the resynchronization of extra-SCN oscillators takes longer than the SCN [278, 279]. Thus, experiencing frequent changes in the light-dark schedule may not allow extra-SCN regions to catch up.

Siberian hamsters are used as a unique model for studying the effects of persistent desynchronization after a phase shift. Siberian hamsters are unique in that they do not readily entrain to a 5 h phase delay. When Siberian hamsters are exposed to a phase-advancing light pulse followed by a phase delay in the photocycle on the subsequent day, they become arrhythmic [280]. Arrhythmic, socially isolated, aged hamsters had increased depression-like behavior, but decreased anxiety-like behavior [281]. In another study, arrhythmic Siberian hamsters showed impaired novel object recognition and spatial memory [282]. Circadian gene expression rhythms were arrhythmic in the SCN of behaviorally arrhythmic hamsters, and lesioning the SCN rescued the spatial and recognition memory of the hamsters, suggesting that a dysfunctional SCN caused the cognitive and behavioral disturbances in the arrhythmic hamsters [282, 283]. However, it is unknown whether lesioning the SCN normalizes their depression- and anxiety-like behaviors, which would indicate that an arrhythmic, intact SCN causes mood-like disturbances. Since mood-like behaviors of young hamsters were not affected and circadian rhythms become less robust with age, this suggests that circadian disturbances have greater effects on mood in older individuals or vulnerable populations [281, 284].

### 6.4. T Cycles (Non-24 h Cycles)

T cycle studies have yielded mixed conclusions on whether circadian rhythm disruptions may explain the effects of light on mood. In a study by Karatsoreos and colleagues, mice exposed to a T20 schedule (LD 10 : 10) had reduced anxiety-like behavior, cognitive flexibility, and dendritic complexity in the medial prefrontal cortex [285]. The T20 schedule did not affect overall sleep, but activity rhythms and sleep timing, thus showing an association between circadian rhythm disruptions and disturbed anxiety and cognitive-related behaviors [286]. In a study by LeGates and colleagues, exposure to a T7 schedule (LD 3.5 : 3.5) slightly increased the period of body temperature and activity rhythms of mice [18]. Exposure to the T7 cycle also increased depression-like behavior, but had no effect on anxiety-like behavior. Since no difference was found in SCN *Per2* expression rhythms, and the mice maintained rhythmic locomotor activity, it was concluded that T7 cycle-induced mood-like disruption was likely due to an SCN-independent mechanism. A third study by Ben-Hamo and colleagues showed that rats exhibit increased immobility in the forced swim test and decreased sexual behavior after exposure to a T22 cycle (LD 11 : 11) [238]. Previously, it was shown that rats under a T22 cycle displayed two different locomotor activity rhythms with two different periods [287]. This was attributed to a desynchronization of SCN molecular rhythms, since the ventral and dorsal SCN of T22-exposed rats had a desynchronized expression of circadian genes [287]. Thus, together, these T cycle studies emphasize that light may affect mood through multiple mechanisms [266].

## 7. The SCN in Animal Models of Mood Disorders

### 7.1. Stress-Related Models

Evidence supports that stress is a risk factor for depression [288, 289]. In rodents, chronic stress can produce behaviors resembling depression in humans, and these depression-like behaviors are responsive to antidepressant treatment [290]. In rodents, chronic stress disrupts body temperature, activity, and hormone rhythms [291–294]. Thus, there is interest in determining whether chronic stress disrupts SCN function, which may explain some of the effects of chronic stress. Some studies show that chronic stress paradigms dampen PER1, PER2, CLOCK, or BMAL1 expression rhythms in the SCN [292, 295–297]. Specifically, in a study from our lab, unpredictable chronic mild stress (UCMS) desynchronized the phase of clock gene expression rhythms in the SCN of mice [292]. Furthermore, SCN PER2::LUC amplitude was greatly reduced following UCMS. Most notably, SCN PER2::LUC amplitude was positively correlated with swim time in the forced swim test and open arm time in the elevated plus maze, indicating that UCMS-mediated disruption of the molecular clock in the SCN was directly associated with the severity of depression- and anxiety-like behaviors of the mice.

On the other hand, there are studies showing no effect or opposite effects of stress paradigms on PER expression rhythms in the SCN of rodents [298–300]. These conflicting results may be related to the type of stressor (e.g., social defeat stress, learned helplessness), different paradigm durations, and the timing of the stressors. Furthermore, since previous studies have focused on determining the effects of stress on the core molecular clock in the SCN, these studies may have missed stress effects on SCN neural activity. Just because stress has no effect on the molecular clock in the SCN does not necessarily mean that stress has no effect on SCN output. However, another interpretation is that the SCN is more resilient to the effects of stress. Thus, more work is needed to determine if SCN neuronal rhythms are disrupted in chronically stressed mice, and if the SCN plays a role in the mood-like phenotype of chronically stressed rodents.

### 7.2. Sleep Deprivation Models

Since a few studies report that sleep deprivation can trigger hypomania or mania in bipolar disorder, researchers have used sleep deprivation paradigms to model mania-like behaviors in rodents [301, 302]. Typically, sleep deprivation is induced using the platform model, where rodents are placed on a small platform surrounded by water [303]. If the animal falls asleep, it will fall in the water. Thus, rodents will stay awake. Investigators typically sleep deprive rodents for 72 hrs. After the sleep deprivation period, the animals show mania-like behaviors such as insomnia, hyperactivity, aggression, stereotypy, and hypersexuality (as reviewed in [304]). Lithium and antipsychotics prevent some of the effects of sleep deprivation, indicating that the model has predictive validity [303, 305].

Sleep deprivation is known to affect circadian gene expression. Sleep deprivation increases *Per1–3* expression [306–308]. More recently, Curie and colleagues showed that sleep deprivation increased PER2 protein levels in the brain, liver, and kidney [309]. Notably, the SCN was resilient to the effects of sleep deprivation on PER2 levels [309]. Since perturbing circadian genes affects sleep homeostasis, it is thought that sleep deprivation-induced changes in circadian gene expression are involved in identifying sleep need (as reviewed in [310]). It is important to note that these studies looked at the effects of shorter periods of sleep deprivation than is typically used to induce mania-like behaviors, thus longer sleep deprivation periods may have greater effects on circadian gene expression. Sleep deprivation-induced changes in circadian gene expression in mood-related brain regions may underlie the effects of sleep deprivation on affect. Although the SCN may be resilient to the effects of sleep deprivation on molecular rhythms, studies do indicate that sleep deprivation affects SCN neural activity. Sleep deprivation dampens SCN neural activity and attenuates light-induced increases in SCN neural activity [311, 312]. Moreover, sleep deprivation attenuates the photic phase-shifting of locomotor activity [312, 313]. Furthermore, as previously discussed, behavioral arousal during the rest period increases serotonin in the SCN and induces large phase advances during the day in nocturnal animals [132, 314].

Shorter periods of sleep deprivation are antidepressant (as reviewed in [315]). Sleep deprivation also produces antidepressant-like effects in some animal models [316, 317]. Unfortunately, the effects of sleep deprivation are short lived in humans. Although, there is evidence to support that combining sleep deprivation therapy with bright light therapy, sleep phase advance, and medication produces long-lasting antidepressant effects in some individuals [318]. Since these treatments can all affect circadian rhythms, it is possible that combining the treatments increases the likelihood of realigning circadian rhythms in depressed patients.

### 7.3. DAT-KD Mice

A prominent theory is that manic episodes in bipolar disorder are associated with a hyperdopaminergic state. Support for this theory was originally based on studies showing that psychostimulants produce some mania-like symptoms, whereas antidopaminergic drugs reduce manic symptoms (as reviewed in [319]). More recently, neuroimaging studies support that in hypomania there is increased activation of brain regions that are targets of reward-related dopamine projections [319, 320]. A study by Anand and colleagues suggested that reduced dopamine transporter (DAT) availability could explain the increase in reward-related circuitry in hypomania, but this has not been replicated by other studies [321].

To determine the behavioral and neurobiological consequences of reduced DAT availability, researchers developed DAT knockdown (DAT-KD) mice, with ~90% knockdown of DAT. DAT-KD mice exhibit hyperactivity, stereotypy, reduced spatial d (more linear movements), increased risk-taking, and increased motivation for reward, but no sensorimotor deficits in prepulse inhibition tests, indicating that DAT-KD mice display many mania-like behaviors [322–326]. Moreover, valproic acid reduced hyperactivity in DAT-KD mice, suggesting that some of the behaviors of DAT-KD mice are responsive to mood stabilizers [327]. Although these mice have been primarily considered a useful model of mania-like behaviors, elevated dopamine signaling and many of the behaviors of DAT-KD mice are relevant for studying other psychiatric disorders such as attention-deficit/hyperactivity disorder and schizophrenia [326].

Circadian rhythms are also disrupted in DAT-KD mice. DAT-KD mice have a longer activity period and increased sensitivity to photic phase delays [183]. Surprisingly, SCN PER2::LUC rhythms from wildtype and DAT-KD mice were indistinguishable. Landgraf and colleagues did observe lengthening of SCN PER2::LUC rhythms after application of a D1R agonist. Thus, they concluded that the SCN explants had lost afferent dopaminergic projections, which may explain why PER2::LUC rhythms were not affected in DAT-KD mice. Chronic valproic acid treatment, using the same administration paradigm found to reduce hyperactivity in these mice, shortened the wheel-running period of the DAT-KD mice [183, 327]. Wheel-running rhythms then lengthened upon removal of valproic acid. When applied to SCN slices, valproic acid shortened PER2::LUC period, but at a higher concentration than needed to affect wheel-running rhythms. Interestingly, valproic acid shortened PER2::LUC rhythms in a mouse hippocampal cell line at lower concentrations, suggesting that extra-SCN regions are more sensitive to the period shortening effects of valproic acid and mediate the effects of valproic acid on wheel-running rhythms. Thus, if SCN rhythms are disrupted in DAT-KD mice, it may not greatly contribute to the mania-like phenotype of DAT-KD mice since valproic acid affected hyperactivity and wheel-running rhythms at doses that did not alter PER2::LUC rhythms in the SCN. However, it is possible that at lower doses, valproic acid affected SCN neural activity rhythms or other molecular targets in the SCN. Thus, it would be interesting to look in more detail at the effects of DAT-KD and valproic acid on SCN rhythms.

### 7.4. *Myshkin* Mice

*Myshkin* mice were selected in an epilepsy-like phenotype-driven *N*-ethyl-*N*-nitrosourea (ENU) mutagenesis screen [328]. When backcrossed for 20 generations onto a C57BL/6NCr strain, these mice no longer exhibited stress-induced seizures, but showed mania-like behaviors [329]. Heterozygous *Myshkin* mice (*Myk*/+) show hyperactivity in the open field, increased object exploration, increased risk-taking behavior in the elevated plus maze and light/dark box, and increased reward seeking [329]. Moreover, lithium and valproic acid produce therapeutic-like effects in *Myk*/+ mice, reducing hyperactivity and risk-taking behavior [329]. *Myk*/+ mice carry a missense mutation in the *Atp1a3* gene, encoding the neuron-specific *α*3 subunit of Na^+^/K^+^-ATPase [328]. The mutation reduces Na^+^/K^+^-ATPase activity and increases glutamate-evoked Ca^2+^ signaling in cortical neurons, suggesting that the mutation increases neuronal excitability [328]. Studies have implicated *ATP1A3* in bipolar disorder, further supporting the translational utility of these mice [330, 331].

Since *Atp1a3* is expressed in the SCN and circadian rhythm disturbances frequently occur in mania, Timothy and colleagues looked at the wheel-running rhythms of *Myk*/+ mice [332]. *Myk*/+ mice had longer wheel-running periods and active phases. *Myk*/+ mice also had dampened wheel-running rhythms, with some *Myk*/+ becoming arrhythmic in constant conditions. To determine if perturbations in the SCN could explain the circadian phenotype of *Myk*/+ mice, Timothy and colleagues looked at SCN PER2::LUC rhythms and neural activity. While there was no difference in SCN PER2::LUC rhythms, whole-cell current-clamp recordings revealed that the *Myk* mutation resulted in a loss in the day/night change in SCN spontaneous firing frequency. Thus, indicating the *Myk* mutation dampens SCN neural activity rhythms. *Myk*/+ mice were also more sensitive to photic phase delays and did not show a typical phase advance in response to light pulses late in the active phase, suggesting that the SCN of *Myk*/+ mice may respond differently to ipRGC input [332]. Indeed, the SCN of *Myk*/+ mice showed greater AMPA-evoked increases in intracellular Ca^2+^, supporting that the SCN of *Myk*/+ mice is more sensitive to glutamate from ipRGCs during the early subjective night.

Thus, Na^+^/K^+^-ATPase can regulate both mania-like behaviors and circadian rhythms. The findings by Timothy and colleagues indicate that the circadian phenotype of *Myk*/+ mice is likely mediated by a loss of Na^+^/K^+^-ATPase in the SCN, but the brain regions underlying the mood-related effects of the *Myk* mutation are unclear [332]. Thus, brain region-specific manipulations of the Na^+^/K^+^-ATPase *α*3 subunit may be worthwhile to determine whether the circadian and mania-like phenotypes of *Myk*/+ mice are due to pleiotropic effects of the *Atp1a3* gene.

### 7.5. Circadian Gene Models

#### 7.5.1. *Clock*Δ19 Mice

*Clock*Δ19 mice were selected in a circadian phenotype-driven ENU mutagenesis screen [333]. *ClockΔ*19 mice carry a point mutation that results in the removal of exon 19 during gene splicing, which leads to a dominant-negative CLOCK protein [334]. Heterozygous *ClockΔ*19 mice exhibit greater photic phase delays and advances relative to wildtype mice [335]. In constant darkness, homozygous *Clock*Δ19 mice have a longer activity period and eventually become arrhythmic [333]. In the SCN, the *Clock* mutation lengthens the neuronal firing period and dampens *Per* expression [335, 336]. Similarly, overexpressing *Clock*Δ19 in neuromedin S-positive cells, where neuromedin S is a peptide that is highly expressed in the SCN, lengthened running wheel and SCN PER2::LUC rhythms [337]. Thus, the circadian phenotype of *Clock*Δ19 mice is likely due to disrupted *Clock* function in the SCN.

It is well established that *Clock*Δ19 mice display many mania-like behaviors, including hyperactivity in the open field, increased risk-taking in the elevated plus maze and light/dark box, reduced immobility in the forced swim test, and increased reward seeking [338–340]. *Clock*Δ19 mice also display mood-like cycling with increased mania-like behaviors during the day and returning to a euthymic-like state during the night [340].

Work from our lab suggests that CLOCK in the VTA plays a primary role in mood regulation. Knocking down CLOCK in the VTA is sufficient to produce mania-like hyperactivity and increased risk-taking behavior, whereas expressing functional CLOCK in the VTA of *Clock*Δ19 mice normalizes the locomotor and risk-taking behavior to wildtype levels [339, 341]. During the day, when *Clock*Δ19 mice display mania-like behaviors, tyrosine hydroxylase expression was elevated, as well as the firing rate of VTA dopaminergic neurons [340, 342]. Inhibiting tyrosine hydroxylase activity during the day reversed the mania-like behaviors of *Clock*Δ19 mice [340]. Moreover, optogenetic stimulation of VTA dopaminergic neurons during the day induced hyperactivity and more risk-taking behavior [340]. Together, this work indicates that elevated VTA dopaminergic neuron signaling underlies the hyperactivity and risk-taking behavior in *Clock*Δ19 mice.

Overall, pharmacological and brain-region specific studies in the lab indicate that the effects of the *Clock*Δ19 mutation on circadian rhythms and mood are likely pleiotropic effects. In a study by Arey and colleagues, a casein kinase 1 inhibitor, which is known to increase circadian period, reduced the risk-taking behaviors of *ClockΔ*19 in the elevated plus maze and light/dark box [343]. Thus, the casein kinase 1 inhibitor likely exacerbated the longer period of *Clock*Δ19, but still reduced their risk-taking behaviors. Furthermore, knocking down CLOCK in the VTA increased locomotor activity and produced risk-taking behavior, but decreased circadian period, opposite of *Clock*Δ19 mice [341]. Together, these studies indicate that the increased risk-taking behaviors induced by impaired CLOCK function are not dependent upon having a long circadian period.

#### 7.5.2. *Rev-erbα* KO Mice

Preclinical studies have shown that REV-ERB*α* regulates a multitude of mood-related behaviors. *Rev-erbα* KO mice show reduced immobility in the forced swim and tail suspension tests, increased risk-taking behavior in the elevated plus maze, hyperactivity, elevated aggression, and increased motivated behaviors [344, 345]. REV-ERB*α* is not necessary to maintain circadian rhythms, as REV-ERB*β* appears to have some overlapping functions [346]. However, *Rev-erbα* KO mice do have a shorter circadian period, increased sensitivity to photic phase advances, and a reduced amplitude of *Bmal1* expression in the SCN [347].

Although loss of REV-ERB*α* affects SCN molecular rhythms, the aberrant mood-like behaviors in *Rev-erbα* KO mice appear to be due to the actions of REV-ERB*α* outside of the SCN. Specifically, *Rev-erbα* KO mice have elevated tyrosine hydroxylase levels in the substantia nigra and VTA as the result of loss of direct repression of tyrosine hydroxylase transcription by REV-ERB*α* [345]. Pharmacological inhibition of tyrosine hydroxylase reduced the locomotor activity of the *Rev-erbα* KO mice to wildtype levels [345]. Moreover, pharmacological inhibition of REV-ERB*α* in the ventral midbrain recapitulated the mania-like behaviors of *Rev-erbα* KO mice [345]. Together, these experiments suggest that loss of REV-ERB*α* in the ventral midbrain largely explains the mania-like behavior of *Rev-erbα* KO mice. Interestingly, as a nuclear receptor, REV-ERB*α* is a druggable circadian molecular target. Thus, REV-ERB agonists may be useful for treating mood disorders, if their side effects can be overcome.

#### 7.5.3. FBXL3 and CRY

FBXL3 is a member of the F-box and Leu-rich repeat family of E3 ubiquitin ligases. A mutation in *Fbxl3* (*After hours*, *Afh*) was discovered by an ENU mutagenesis screen to result in a long circadian activity period [348]. SCN PER2::LUC rhythms are also delayed and dampened in *Afh* mice [348]. Moreover, *Afh* mice are more sensitive to photic delays and advances [349]. In terms of mood-related behaviors, *Afh* mice display reduced depression-like behavior and increased risk-taking, similar to *ClockΔ*19 mice [350]. FBXL3 promotes the ubiquitination and proteasomal degradation of CRY [351]. Thus, the *Afh* or *Fbxl3* mutation results in an upregulation and stabilization of CRY, which underlies the period lengthening effects of *Afh* [321, 352]. Further supporting that FBXL3 regulates circadian period through CRY, a mutation in *Cry2* that enhances FBXL3 binding to CRY2 resulted in a shortened activity period in mice [353].

*Cry* knock-out mice studies support that *Cry* modulates mood-related behaviors. Specifically, *Cry1^−/−^* mice have increased depression-like behavior, *Cry2^−/−^* mice have reduced sucrose preference, and *Cry1^−/−^;Cry2^−/−^* double knock-out mice have increased anxiety-like behavior [354–356]. Together, these studies suggest that abnormally reduced FBXL3-mediated destabilization of CRY may result in a longer period and mania-like behavior. Conversely, increased FBXL3-mediated destabilization of CRY may result in a shorter period and depression-like behavior. However, not all studies support this model. For example, *Cry2^−/−^* mice have a lengthened period, suggesting that the effects of these genes on the central clock are dissociable from the effects on mood-like behaviors [321].

#### 7.5.4. Per Mice

Mice expressing mutant *Per2* (*Per2^Brdm1−/−^* mice), which have a deletion incorporating the PAS domain that is important for protein/protein interactions, display some mania-like behaviors [357]. *Per2^Brdm1−/−^* mice show reduced immobility in the forced swim test, increased sensitivity to cocaine, and elevated alcohol consumption [358–360]. However, loss of functional *Per2* does not result in consistent effects on anxiety-like behavior [361]. A different *Per2* mutant mouse (*Per2^ldc^*), which also has a deletion that incorporates the PAS domain, was found to have inconsistent anxiety-like behaviors across measures in the elevated plus maze and light/dark box [361]. Although when crossed with mice that lack functional *Per1*, the double mutant mice showed increased anxiety-like behavior, indicating that disrupting both genes is necessary to perturb anxiety-like behavior [361]. Together, this work suggests that the positive and negative arms of the molecular clock have opposite roles in the regulation of anxiety-like behavior. *ClockΔ*19 mice show decreased anxiety-like behavior, whereas loss of functional PER1 and PER2 has the opposite effect [339]. Thus, it would be interesting to examine other mood-like behaviors of *Per1^ldc^*/*Per2^ldc^* double mutant mice to determine if the opposing roles of CLOCK and PER only apply to anxiety-like behaviors.

More recently, *Per3* was shown to regulate mood-like behavior in rodents. Zhang and colleagues assessed the depression-like behaviors of *Per3* knockout mice (*Per3^−/−^*) and *hPER3-P415A/H417R* transgenic mice [362]. *hPER3-P415A/H417R* was generated to express human PER3 with two missense variants that had been identified to cause familial advance sleep phase that is associated with increased depression and seasonality symptoms. Interestingly, both *Per3^−/−^* and *hPER3-P415A/H417R* showed increased immobility in the tail suspension tests. The missense mutations decreased PER3 protein levels and PER3 repressor activity, indicating that loss of functional PER3 increases the risk for depression.

The stability and localization of *Period* genes in the SCN sets the oscillating speed of circadian rhythms. Loss of either *Per1* or *Per2* leads to period shortening and subsequently arrhythmia in constant conditions [357, 363]. *Per3* is thought to play a lesser role in regulating the central pacemaker, as *Per3^−/−^* mice exhibit only a slightly shorter free-running period and slightly shorter period of PER2::LUC rhythms in the SCN [364, 365]. Interestingly, a recent study by Shi and colleagues identified another missense mutation in *hPER3* (*hPER3-P856A*) that is associated with MDD, but slightly lengthens circadian period [366]. Thus, increased or decreased PER3 transcriptional activity may increase risk for MDD. Since the effect on circadian period was small, Shi and colleagues concluded that any mood-related effects of *hPER3* variants were likely due to changes in clock-controlled genes as opposed to SCN timing [366]. Further supporting a role for *Per* genes outside of the SCN in the modulation of mood and anxiety-like behaviors, Spencer and colleagues showed that knocking down both *Per1* and *Per2* in the nucleus accumbens was sufficient to increase anxiety-like behavior in mice [361]. Thus, although *Per* genes regulate both the central clock and mood-related behaviors, these can be independent effects.

## 8. Conclusions and Future Directions

### 8.1. Circadian Rhythm Disturbances in Humans with Mood Disorders

Many studies point to disrupted circadian rhythms in MDD and bipolar disorder. Some of the most consistent findings are an evening chronotype in mood disorders, dampened body temperature rhythms in depression, and delayed rhythms in depression [50, 53, 55, 81]. Notably, there is even postmortem evidence that molecular rhythms are disrupted in mood-related brain regions in mood disorders [67]. Perturbations in circadian gene expression may underlie rhythmic disturbances in some individuals. Numerous studies have implicated circadian genes in mood disorders, but many of the genetic findings have not been replicated [68, 69]. It is possible that targeted studies looking at genetic associations with depression or bipolar disorder in patients with similar circadian disruptions will move the field forward. Several recent large GWAS studies have identified associations between genetic loci near circadian genes with chronotype [367–369]. In addition, mutations in PER2 have been linked with advanced sleep phase syndrome [370], while mutations in CSNK1D and CRY1 have been linked to delayed sleep phase syndrome [371, 372]. Thus, together, these studies indicate that links can be identified between chronotypes and circadian gene perturbations. Since it can be challenging to objectively determine chronotype in subjects with mood disorders, researchers are also studying rhythm disturbances by looking at molecular rhythms in patient-derived fibroblasts [178, 179].

While there is convincing evidence that molecular rhythms are disrupted, it is less clear if the SCN is affected in mood disorders. Studies have implicated AVP, nitric oxide, and melatonin signaling in the SCN in mood disorders, but it is unknown whether SCN neural activity is altered in MDD or bipolar disorder [98–100]. This is a challenging question to address given that the SCN is small and lies deep within the brain. Some studies have imaged the SCN in humans, indicating that it may be possible to identify differences in SCN responsiveness to circadian challenges in subjects with mood disorders [373, 374].

### 8.2. Effects of Pharmacotherapies on the SCN

It is well established that SSRIs, lithium, and agomelatine influence circadian rhythms, suggesting that these drugs could correct rhythm disturbances in individuals with mood disorders. The most robust findings being that lithium lengthens circadian period, SSRIs induce phase advances, and activation of melatonin receptors induce phase shifts at dawn and dusk [131, 137, 153, 197, 198]. Studies suggest that the circadian-related effects of SSRIs, lithium, and agomelatine are due to their actions in the SCN [137, 153, 199]. Landgraf and colleagues recently showed that valproic acid shortens circadian rhythms [183]. In future studies, it will be important to replicate Landgraf and colleagues' finding and uncover the mechanisms underlying the circadian- and mood-related effects of valproic acid.

From our understanding, there is little known about the effects of other classes of drugs on SCN rhythms. Haloperidol was shown to increase *Per1* expression in the SCN, suggesting that antipsychotics affect SCN molecular rhythms [375]. However, if antipsychotics influence the central clock, it is likely through indirect mechanisms, since D1R seems to mediate the effects of dopamine in the SCN [183, 376]. Low-dose ketamine, a promising rapid-acting antidepressant treatment strategy, may also affect the SCN since glutamatergic signaling in the SCN is crucial for photic entrainment. Ketamine was found to dampen circadian gene expression amplitude in mouse embryonic fibroblasts [377]. Thus, studies looking at the effects of other classes of drugs on SCN rhythms may help elucidate the mechanisms underlying the associations of circadian disruptions with mood disorders.

### 8.3. Insights from Studies in Animal Models

**Figure 2:**
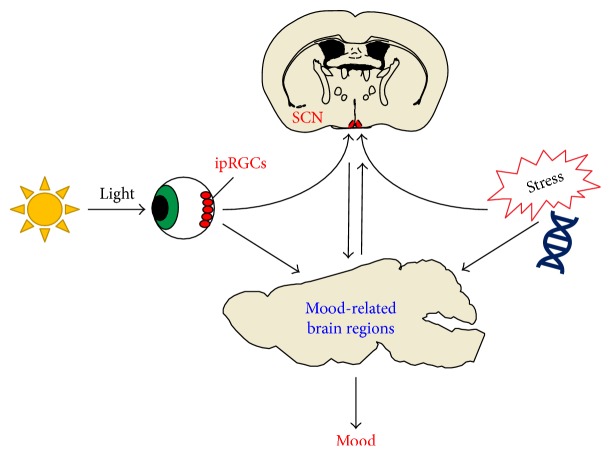
Potential mechanisms underlying the associations between circadian rhythm disruptions and mood disorders. Seasonal changes, jet lag, and shift work may disturb mood in vulnerable individuals through projections from intrinsically photosensitive retinal ganglion cells (ipRGCs) directly to mood-related brain regions, or to the suprachiasmatic nucleus (SCN). Alternatively, other environmental insults (e.g., stress) and genetic disturbances (e.g., circadian gene mutations) can affect mood-related brain regions and SCN function. The SCN may disturb mood by directly or indirectly affecting the function of brain regions more closely tied to mood regulation, explaining how circadian rhythm disturbances could affect mood. Conversely, environmental and genetic factors may influence the SCN and mood-related brain regions independently, explaining how circadian rhythm disturbances could be a noncausal symptom of mood disorders.

Preclinical studies looking at the effects of circadian gene disruption and environmental disturbances support a complex relationship between circadian rhythms and mood (Figure 2). There is evidence to support that disrupting SCN function can causes mood-like disturbances, light can disrupt mood independent of the SCN, and that circadian rhythm disruptions are a noncausal symptom.

The most compelling evidence that the SCN regulates mood comes from the study by Landgraf and colleagues showing that disrupting molecular rhythms in the SCN causes depression- and anxiety-like behaviors in mice [240]. Lesion studies also suggest that a disrupted intact SCN rather than loss of the SCN can lead to aberrant behaviors [239, 282]. If the SCN plays a key role in mood regulation, this leads to many further questions. Do certain types of SCN disruptions cause mood changes? As discussed, genetic perturbations in mice can produce period changes, amplitude reductions, increased sensitivity to photic phase shifts, and mood-like disturbances [183, 327, 329, 332, 333, 335, 339]. Importantly, shifted rhythms, dampened rhythms, and light hypersensitivity have been found in some individuals with mood disorders [50, 88, 91]. In future work, it will also be important to determine which SCN circuits and cell types affect mood. With optogenetic and cell type-specific genetic tools, these more specific questions about the role of the SCN in mood regulation will likely be answered in the coming years.

Light cycle manipulation studies suggest that light regulates mood through multiple mechanisms, both dependent and independent of the SCN. Long and short photoperiods, constant light conditions, shifting light-dark schedules, and various T cycles can all affect mood-like behaviors in animal models. Notably, some of these light schedules, like T7 cycles, do not greatly disturb behavioral circadian rhythms or circadian gene expression, indicating that disrupting SCN function is not necessary for light to affect mood [18]. Moreover, it is known that ipRGCs can directly project to mood-related brain regions, indicating that there are circuits through which light could affect mood independent of the SCN (as reviewed in [378]).

Lastly, animal model studies argue that circadian rhythm disruptions can be an independent symptom of mood disorders. Studies disrupting the expression or function of circadian genes show that circadian genes can regulate circadian rhythms and mood-like behaviors independently. For example, knocking down *Clock* expression in the VTA replicated many of the mania-like behaviors of *ClockΔ*19 mice, but shortened the circadian period, opposite to what is observed in *Clock*Δ19 mice [341]. Thus, the effects of *Clock* on mood-like behavior is dissociable from the effects of *Clock* on the period length of circadian rhythms. Moreover, genetic animal model studies suggest that there are not directional associations between the speed of circadian rhythms and mood-like behaviors. For example, *Rev-erbα* KO mice and *Clock*Δ19 both exhibit mania-like behaviors, but display opposite changes in period length [333, 339, 345, 347]. Furthermore, since both SSRIs (antidepressant) and valproic acid (antimanic) shorten rhythms, the effects of pharmacological treatments on circadian period may not be correlated with their primary effects on mood [56, 183].

### 8.4. Summary

The literature highlights that there are multiple mechanisms that may explain the associations between rhythm disruptions and mood disturbances, one of those mechanisms being disrupted SCN function (Figure 2). We theorize that a disrupted SCN could affect mood by causing internal desynchronization across mood-related brain regions. However, we acknowledge that light could affect mood independent of the SCN through ipRGCs projecting directly or indirectly to mood-related brain regions. Thus, light, other environmental factors, or genetic perturbations may influence mood independent of the central clock.

Overall, these studies suggest that circadian-based therapeutics could treat specific populations of patients with circadian and mood disturbances. With the ability to measure patients' molecular and behavioral rhythms, in addition to detection of genetic polymorphisms, the field is on the cusp of identifying biomarkers for specific subpopulations with circadian and mood disturbances. Animal studies will continue to be important for elucidating the mechanisms underlying the mood and circadian-related effects of therapeutics, gene disruptions, and environmental disturbances, which may lead to novel treatment strategies for mood disorders.

## Figures and Tables

**Table 1:** Sleep and circadian disturbances in major depressive disorder and bipolar disorder.

Psychiatric disorder	Sleep and circadian disturbances
Major depressive disorder	Reduced latency to REM, increased REM time, and decreased slow-wave sleep [45–48] Advanced hormonal rhythms [48–50] Delayed rhythms or an evening chronotype [51–55] Reduced body temperature amplitude and increased body temperature at night [50, 61, 62] Dampened activity, cortisol, thyroid-stimulating hormone, melatonin, and heart rate rhythms [50, 63–65]

Bipolar disorder	Reduced sleep during mania and hypersomnia or insomnia during depression (as reviewed in [70]) Reduced latency to REM and increased REM density during mania [71–73] Evening chronotype [81–83] Less rhythmic and dampened rhythms [77–80] Phase delayed or phase-advanced sleep-wake, metabolite, hormone, or body temperature rhythms [84–87] Advanced rhythms during mania and/or delayed rhythms during depression [88–90] Increased sensitivity to light-induced melatonin suppression [91, 92]

**Table 2:** Effects of pharmacotherapies for mood disorders on locomotor and SCN rhythms.

Drug	Effects on locomotor activity in rodents	Effects on the SCN in rodents
SSRIs	Phase-advanced locomotor activity [130, 131] Shortened locomotor activity period [141, 142]	Phase-advanced neural activity when applied with L-tryptophan in rats [56] Phase-advanced neural activity [137] Shortened PER1::LUC period [143]

Lithium	Lengthened locomotor activity period [153–155]	Lengthened neural activity period [158] Lengthened PER2::LUC period [153, 156, 159, 160] Increased amplitude of PER2::LUC rhythms [153, 156]

Valproic acid	Shortened locomotor activity period [183]	Phase-shifted PER2::LUC rhythms [160] Increased amplitude of PER2::LUC rhythms [160] Shortened PER2::LUC period [183]

Agomelatine	Phase-advanced rhythms [227] Accelerated re-entrainment [232] Entrained locomotor activity rhythms [228, 233]	Decreased firing rate [230, 231]
